# Outcomes of the Use of Pelvic Drain Post-laparoscopic Appendicectomy for Complicated Appendicitis: A Two-Year Single Centre Audit Experience

**DOI:** 10.7759/cureus.92097

**Published:** 2025-09-11

**Authors:** Arafa Bakr, James Tsejime, Ellie Herniman, Monique J Lucas, Chenai Mandangu, Quadri A Sanni, Sameh Fahmy

**Affiliations:** 1 General Surgery, South Warwickshire University NHS Foundation Trust, Warwick, GBR; 2 Urology, South Warwickshire University NHS Foundation Trust, Warwick, GBR

**Keywords:** audit cycle, complicated acute appendicitis, open and laparoscopic surgery, patient outcome research, pelvic drain, surgery general

## Abstract

Introduction: Complicated appendicitis is defined as perforated appendicitis, periappendicular abscess, or peritonitis (acute inflammation of the peritoneum secondary to infection of the appendix). The use of pelvic drains in patients undergoing laparoscopic appendicectomy for complicated appendicitis emerged as a topic of considerable debate.

Aim: This audit aimed to explore local practice and the outcomes of patients undergoing laparoscopic appendicectomy for complicated appendicitis with and without the use of pelvic drains.

Methods: This retrospective audit was conducted at a district general hospital in the West Midlands, UK, between April 2022 and April 2024. All patients undergoing laparoscopic appendicectomy for complicated appendicitis were identified via theatre records, including children (≥5 years old) and adults. Cases were excluded if they were converted to open surgery or performed during another procedure.

Results: Of the patients included in the audit, 454 (94.5%) underwent laparoscopic appendicectomy, while 24 (5%) patients had open surgeries. A total of 216 (47%) patients had intraoperatively complicated appendicitis. Among these, histology confirmed acute appendicitis in 204 (94.4%) patients, whereas 8 (3.7%) patients had a normal appendix and 2 (0.9%) patients had adenocarcinoma. About 84 (39%) patients had a pelvic drain placed, while 132 (61%) patients did not have a drain. The average hospital stay for patients with drains was 5.2 days (SD ±3.8), compared with 2.7 days (SD ±2.2) for those without drains. Around 13% of patients with drains were readmitted, and 6 (7%) patients developed postoperative collections. In comparison, 52% of patients without drains were readmitted, and 27 (20%) of these had postoperative collections.

Conclusion: The use of pelvic drains can be a cause of significant morbidity for patients; however, case-by-case patient selection is needed to ensure improved outcomes.

## Introduction

The use of abdominal drains in surgery can be traced back to antiquity, with Hippocrates reportedly employing drainage techniques in the management of empyema of the gallbladder [1]. In modern surgical practice, the American Association for the Surgery of Trauma has developed a grading system to classify the severity of acute appendicitis, with complicated appendicitis categorised as grade II or higher [2].

Complicated acute appendicitis refers to inflammation of the appendix associated with perforation, gangrene, intra-abdominal abscess, or generalised peritonitis [3,4]. These complications often result from delayed diagnosis or inadequate treatment of initially uncomplicated cases, frequently due to non-specific clinical presentations [5]. Treatment strategies range from conservative management with antibiotics to surgical intervention such as appendicectomy, with varying implications for recovery time and postoperative morbidity [5].

The use of pelvic drains in patients undergoing laparoscopic appendicectomy for complicated appendicitis has emerged as a topic of considerable debate. With the progressive advancement in surgical techniques, understanding the role of drainage systems has become essential to optimising recovery and minimising complications. This audit discusses the impact of pelvic drains on outcomes of recovery in this patient population, highlighting evidence-based practices and their potential benefits as observed in our district general hospital.

Recent studies have emphasised the need for abdominal drainage in patients with complicated appendicitis undergoing laparoscopic appendicectomy. Liao et al. conducted a retrospective cohort study, which showed that drains could reduce postoperative abscess formation and improve general recovery times [2]. This study aligns with the findings of Abu et al., who performed a systematic review and meta-analysis that reinforced the positive impact of drainage placement, particularly by reducing complications associated with fluid collection postoperatively [6]. In addition, Pakula et al. explored the role of drains in laparoscopic procedures. They described the significant decrease in hospital stay attributed to the timely intervention of pelvic drainage. Their research indicates that patients with established intra-abdominal infections may benefit from this approach, corroborating the importance of including preventive measures in surgical protocols [7].

On the other hand, some studies argue that the benefits of postoperative drains may not justify their routine use. For example, Abdulhamid and Sarker questioned the economic implications and effectiveness of routine drainage after emergency appendicectomy, raising concerns about complications related to the drain itself [8]. Additional research, such as the one conducted by Fadl et al., provides information on the lack of drainage in some instances, suggesting that the decision should be adapted to the needs and circumstances of individual patients [9]. In addition, Martinez-Perez et al. explored predictors for a prolonged time of postoperative hospital stay, indicating that although drains can help in specific cases, they do not apply universally to all patients who suffer complicated appendicitis [10].

In summary, using pelvic drains for patients with complicated appendicitis undergoing laparoscopic appendicectomy may benefit some patients; the decision to employ this practice should be individualised. The existing literature has an attractive argument for using drains to facilitate recovery and avoid complications; however, the clinical scenario of each patient should guide this practice, ensuring that unnecessary interventions are avoided. More studies are necessary to strengthen the basis of evidence around abdominal drainage in this context.

## Materials and methods

Study design

This audit was a single-centre retrospective study based in a district general hospital in the United Kingdom. The patient data were selected from April 2022 to April 2024.

The eligibility criteria were created to include all patients in this time period who underwent emergency laparoscopic appendicectomy with the three-port technique. Patients were excluded if they had an appendicectomy that used an open technique, as well as if the appendicectomy was a part of another procedure. In our hospital, patients are accepted from 5 years of age and above; those younger are referred to a specialist paediatric service. Procedures were conducted by either consultant general surgeons or senior registrars under consultant supervision. A total of 454 patients underwent laparoscopic appendicectomy, of whom 216 had complicated acute appendicitis.

The following patient demographics were collected from the surgical procedure notes and histological laboratory reports: age, gender, procedure, intraoperative findings such as the presence of inflammation or perforation in the appendix, free pus, gangrene, histological findings and use of antibiotics intra- and postoperatively (including duration of course), use of drain, hospital length of stay, and cause for readmission. 

Data collection

Data were collected retrospectively by extracting patient data from electronic records and sources, including previous operation notes, inpatient notes, prescription charts, and discharge summaries.

## Results

**Figure 1:**
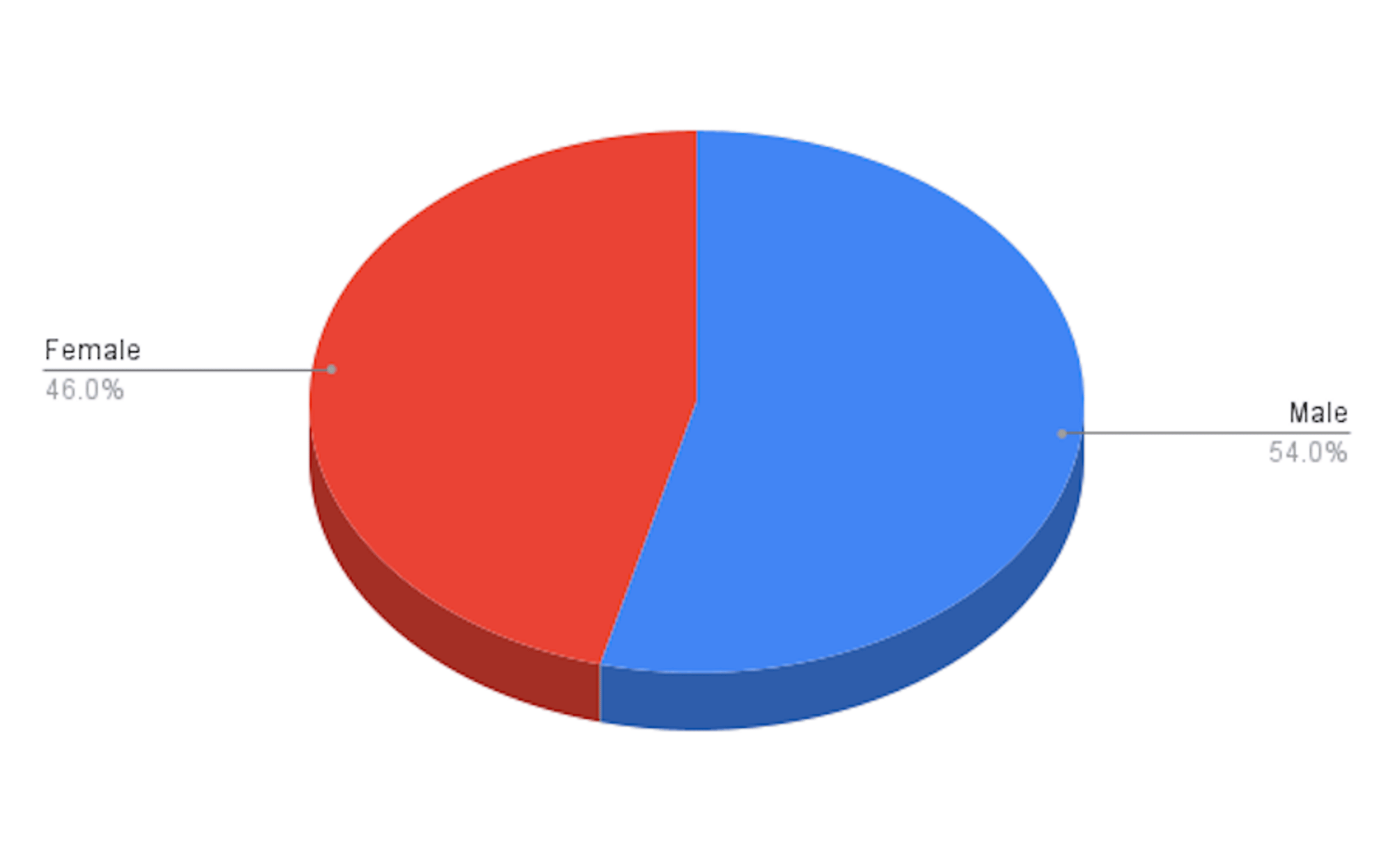
Gender distribution of patients with complicated appendicitis N=216

**Figure 2:**
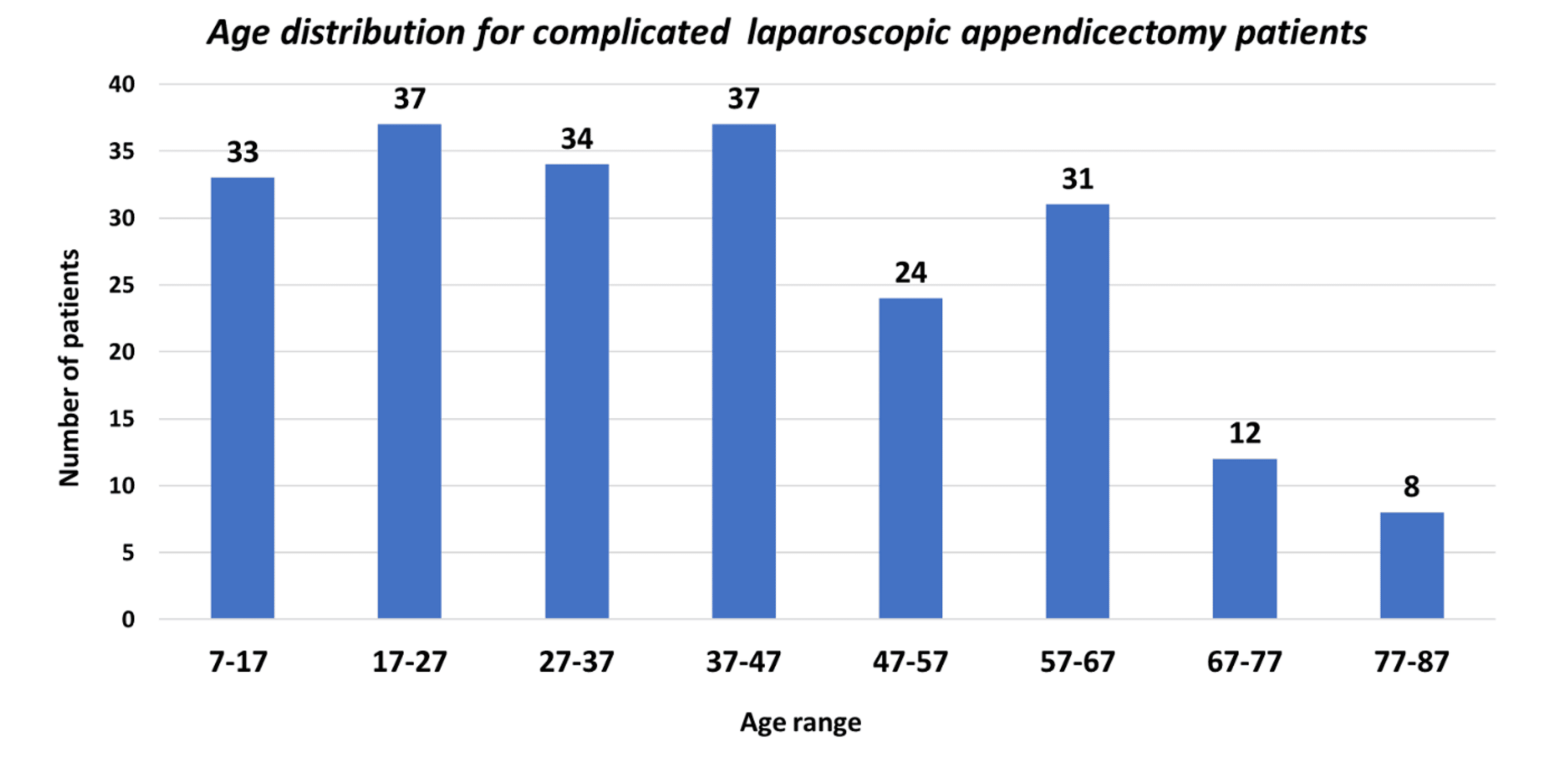
Age distribution of patients with complicated laparoscopic appendectomy Mean age: 29 ± 21.2 years

A total of 216 (48%) patients were diagnosed with complicated acute appendicitis, with 116 males and 100 females (Figure 1). The patients’ ages ranged from 7 to 87 years (Figure 2), with a mean age of 29 ± 21.2 years.

Intraoperative findings

Intraoperative findings, as documented by the operating surgeons, indicated an inflamed appendix in 193 (89%) patients. Of these patients, 127 (58.7%) were reported as perforated, 87 (40.2%) as gangrenous, and free pus was identified in 156 (72.2%) patients (Table 1). Histological examination revealed that 204 (94%) patients had acute appendicitis, and 8 (3.7%) patients had a normal appendix. Perforated diverticulum and adenocarcinoma were found in 2 (0.9%) patients each (Table 2).

**Table 1:** Showing the intraoperative findings N=216

Breakdown of complicated appendicitis findings		
Intraoperative findings	Frequency (n)	Percentage (%)
Perforated appendix	127	58.7
Gangrenous appendix	87	40.2
Free pus	156	72.2

**Table 2:** Showing histological findings in patients who had complicated appendicitis N=216

Histological findings in complicated appendicitis		
Histology	Number of patients (n)	Percentage (%)
Acute appendicitis	204	94.4
Normal appendix	8	3.7
Perforated diverticulum	2	0.9
Adenocarcinoma	2	0.9

Drain usage

**Figure 3:**
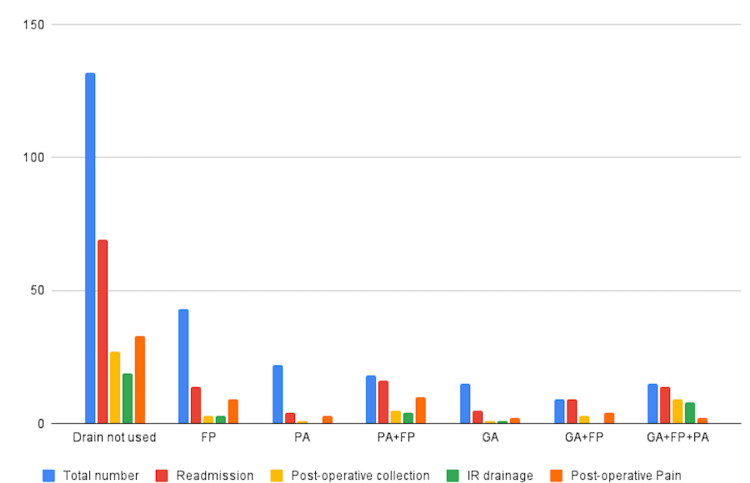
Postoperative complications in the cohort of patients who did not have drain insertion FP, Free pus; GA, Gangrenous appendix; IR, Interventional radiology; PA, Perforated appendix

Eighty-four (38.9%) patients had drains, compared with 132 (61.1%) who did not. The average hospital stay was 5.2 days (SD ±3.8) for patients with drains, compared with 2.7 days (SD ±2.2) for those without drains. A total of 13 (15.5%) patients with drains were readmitted, and 6 (7.1%) patients had postoperative collections, leading to 2 (2.3%) of these patients then undergoing drainage during their second admission (Figure 3). The most common indication for drain insertion is the presence of free pus, as observed in 71 (84.5%) patients.

**Figure 4:**
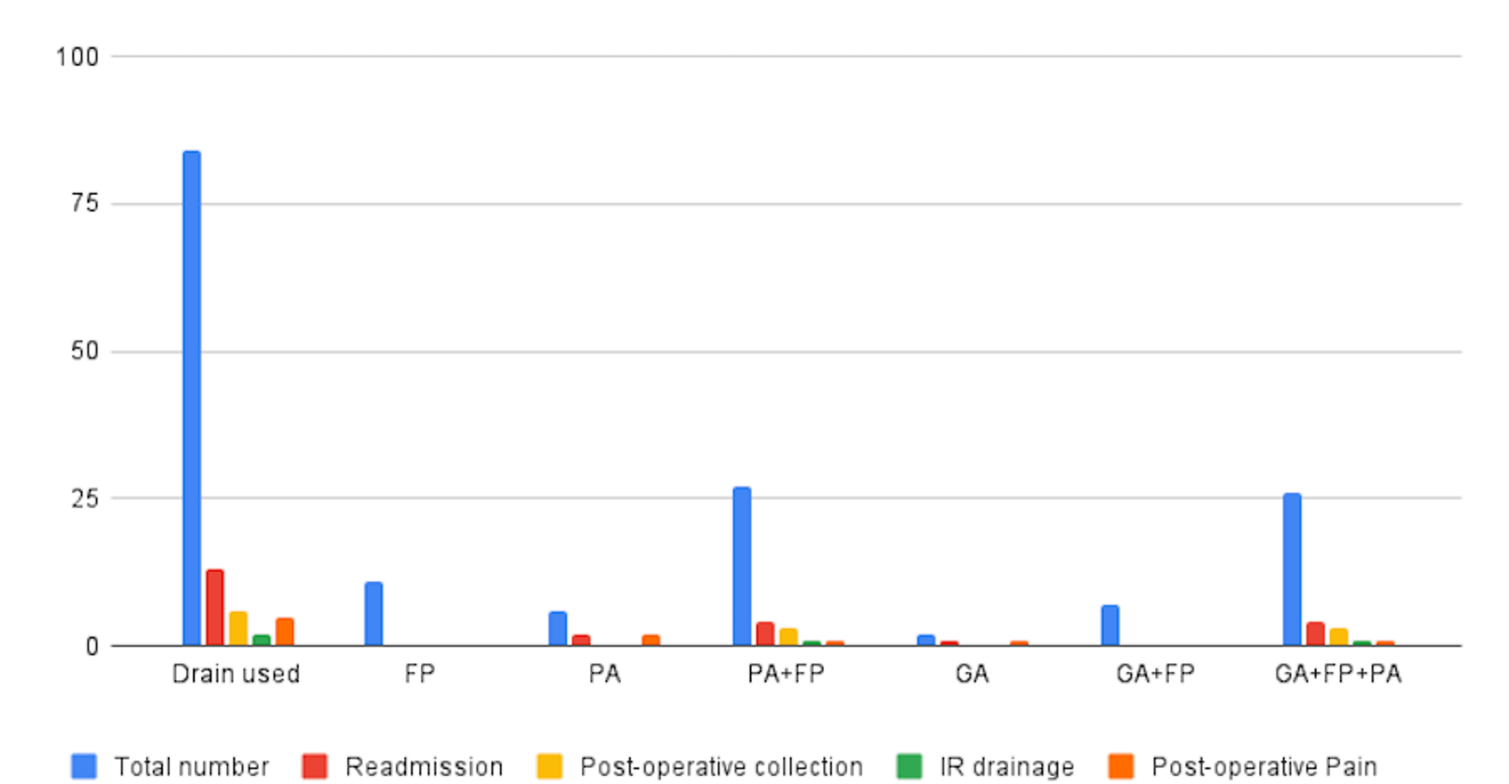
Postoperative complications in the cohort of patients who had initial drain insertion FP, Free pus; GA, Gangrenous appendix; IR, Interventional radiology; PA, Perforated appendix

**Figure 5:**
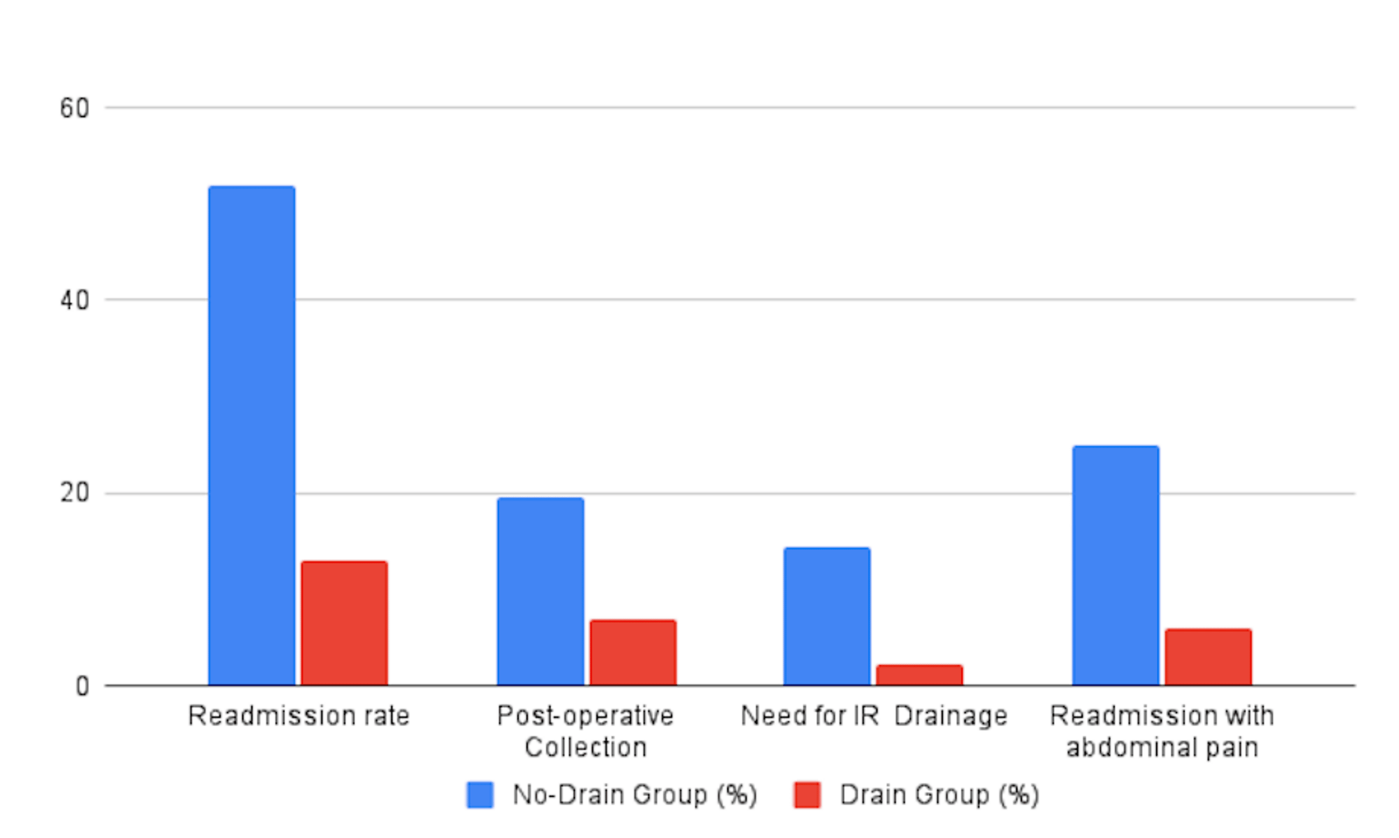
Outcomes of drain insertion versus non-drain insertion IR, Interventional radiology

 In comparison, of the patients without abdominal drainage, 68 (52%) were readmitted, and 27 (39.7%) of these had postoperative collections, resulting in 20 (74.1%) patients undergoing drainage (Figure 4). Figure 5 shows the outcomes in patients who had a drain inserted compared to those who did not have a drain.

A Mann-Whitney U test was used to compare the mean length of stay in patients with or without drain insertion: The z-score was -6.80032. The p-value was <0.00001, giving a statistically significant result at p<0.05.

The Chi-squared formula was used to assess the significance between the drainage versus no-drainage groups on readmission rates. The chi-square result was 33.2074. The p-value was 0.0000, significant at p<0.05. The same formula was used to compare significance in postoperative collection rates and found a chi-square of 9.5739 and a p-value of 0.0020 (significant at p<0.05).

## Discussion

In selected cases, the use of pelvic drains may provide clinical benefits by facilitating the continuous evacuation of contaminated fluid, thus reducing the risk of infection and promoting faster recovery [4]. Although routine drain use after appendicectomy is generally discouraged, selective drainage is considered beneficial in scenarios involving diffuse peritonitis, extensive contamination, large pus collections that are difficult to irrigate completely, risk of appendicular stump fistula, or faecal contamination following perforation [2].

In their systematic review, Abu et al. found that abdominal drainage significantly reduced the incidence of postoperative complications related to intra-abdominal collections [6]. Similarly, Human et al. reported improved outcomes in paediatric patients, suggesting broader applicability of selective drainage across age groups [11]. Collectively, these studies support the strategic use of drains in managing complicated appendicitis, particularly during laparoscopic procedures.

In our two-year retrospective analysis, 47% of patients were diagnosed with complicated appendicitis, a proportion higher than the 27.6% reported by Mahattanobon et al. in a Thai pediatric cohort, but comparable to the 43.95% incidence reported by Piotrowska et al. [12,13].

Drains were primarily placed in patients with free pus, accounting for 32.8% of all cases. Among those who received drains, 13% were readmitted, with an average hospital stay of 5 days. Additionally, 7% developed postoperative intra-abdominal collections, and 2.3% required radiological drainage. These complication rates are notably lower than those reported by Nazarian et al. at Whittington Hospital, UK [14], where 34.6% of patients experienced postoperative complications and 19.2% developed intra-abdominal collections and are consistent with the study by Pakula et al., which reported similar rates of postoperative intervention [7]. However, they contrast with the results of Fadl et al., who found that abdominal drainage did not reduce postoperative complications and was associated with delayed recovery [9]. Our findings also align with those of Sorooshian et al., who evaluated the outcomes of drain usage in acute appendicitis before and after an educational intervention aimed at improving clinician awareness and decision-making [15].

In contrast, the no-drain group had a shorter average hospital stay of 2.8 days but exhibited significantly higher rates of readmission (52%). Of these, 19.6% had postoperative intra-abdominal collection, 25% were readmitted with abdominal pain, while 3.7% presented with symptoms such as fever, nausea, or vomiting. Notably, 14.3% required radiological drainage, approximately double the rate observed in the drain group. These trends are in line with Rather et al., who reported longer hospital stays in the drain group (6.5 days vs. 4 days) [16].

Overall, our two-year review suggests that while the omission of drains may reduce the initial length of hospital stay, it may be associated with increased readmission and complication rates. These data support a selective, case-by-case approach to the use of drains in complicated appendicitis. In high-risk patients, drainage appears to mitigate the likelihood of delayed complications and the need for subsequent interventions. Our observed complication rates (13% readmission, 7% intra-abdominal collections, and 2.3% requiring radiological drainage) compare favourably with those reported by Nazarian et al. and support a potential role of drainage in improving patient outcomes [14].

This study has several limitations, most notably its retrospective design and the potential for selection bias in the decision to place drains. These factors may influence outcomes due to variability in clinical judgment and case severity. To more definitively assess the role of selective drainage in complicated appendicitis, further prospective, randomised controlled trials are warranted. 

## Conclusions

Although the use of pelvic drainage in the management of complicated appendicitis remains a subject of debate among surgeons, our findings suggest that a pelvic drain offers a real clinical benefit in selected cases. Careful patient selection guided by clinical judgment and intraoperative findings is essential to optimise outcomes.

## References

[1] Abdul Jawad K, Urrechaga E, Cioci A (2021). Discordance in appendicitis grading and the association with outcomes: a post-hoc analysis of an EAST multicenter study. J Surg Res.

[2] Liao YT, Huang J, Wu CT (2022). The necessity of abdominal drainage for patients with complicated appendicitis undergoing laparoscopic appendectomy: a retrospective cohort study. World J Emerg Surg.

[3] Skjold-Ødegaard B, Søreide K (2022). The diagnostic differentiation challenge in acute appendicitis: how to distinguish between uncomplicated and complicated appendicitis in adults. Diagnostics (Basel).

[4] Andersen BR, Kallehave FL, Andersen HK (2005). Antibiotics versus placebo for prevention of postoperative infection after appendicectomy. Cochrane Database Syst Rev.

[5] Moris D, Paulson EK, Pappas TN (2021). Diagnosis and management of acute appendicitis in adults: a review. JAMA.

[6] Abu A, Mohamedahmed AY, Alamin A (2022). Evaluation of drain insertion after appendicectomy for complicated appendicitis: a systematic review and meta-analysis. Cureus.

[7] Pakula AM, Skinner R, Jones A (2014). Role of drains in laparoscopic appendectomy for complicated appendicitis at a busy county hospital. The American SurgeonTM.

[8] Abdulhamid AK, Sarker SJ (2018). Is abdominal drainage after open emergency appendectomy for complicated appendicitis beneficial or waste of money? A single centre retrospective cohort study. Ann Med Surg (Lond).

[9] Fadl EMA, Amer AF, Abdelrahim HS (2022). Is abdominal drain necessary after laparoscopic appendectomy for complicated appendicitis?. Surg Gastroenterol Oncol.

[10] Martínez-Pérez A, Payá-Llorente C, Santarrufina-Martínez S, Sebastián-Tomás JC, Martínez-López E, de'Angelis N (2021). Predictors for prolonged length of stay after laparoscopic appendectomy for complicated acute appendicitis in adults. Surg Endosc.

[11] Human MJ, Tshifularo N, Mabitsela M (2022). Laparoscopic appendectomy for complicated appendicitis in children: does the post-operative peritoneal drain make any difference? A pilot prospective randomised controlled trial. Pediatr Surg Int.

[12] Mahattanobon S, Samphao S, Pruekprasert P (2014). Clinical features of complicated acute appendicitis. J Med Assoc Thai.

[13] Piotrowska A, Osman S, Wolak PK (2017). Incidence of complicated acute appendicitis: a single-centre retrospective study. Med Stud.

[14] Nazarian S, Boardman C, Chohda E, Shah A (2021). Abdominal drainage in complicated appendicectomy - resources down the drain?. J Nepal Health Res Counc.

[15] Sorooshian P, Ward R, Sandison A (2022). A simple intervention to improve the use of postoperative antibiotics and intra-abdominal drains in appendicectomy patients. Ann R Coll Surg Engl.

[16] Rather SA, Bari SU, Malik AA, Khan A (2013). Drainage vs no drainage in secondary peritonitis with sepsis following complicated appendicitis in adults in the modern era of antibiotics. World J Gastrointest Surg.

